# Role and Molecular Mechanisms of Aerobic Glycolysis in Gastrointestinal Tumors

**DOI:** 10.7150/jca.125305

**Published:** 2026-01-01

**Authors:** Yulin Lei, Lin Peng, Zedong Qin, Jing Luo, Yani Luo, Zujie Fan, Jianmei Wang, Shike Huang, Huarong Zhao, Sheng Lin, Li Xiang, Yuhao Luo

**Affiliations:** 1 Department of Oncology, The Affiliated Hospital of Southwest Medical University, Luzhou, China.; 2 Department of Bone and Joint Surgery, The Affiliated Hospital of Southwest Medical University, Luzhou, China.; 3 Department of Oncology, The Xichang People's Hospital, Xichang, China.; 4 Department of Cardiology, The Affiliated Hospital of Southwest Medical University, Luzhou, China.; 5 Department of Pathology, The Affiliated Hospital of Southwest Medical University, Luzhou, China.; 6 Department of Oncology, Hejiang County People's Hospital, Luzhou, China.

**Keywords:** aerobic glycolysis, Warburg effect, gastrointestinal tumors, immune cells, tumor microenvironment

## Abstract

Gastrointestinal tumors are among the most common tumors worldwide and are currently the leading cause of cancer-related deaths. Gastrointestinal tumors affect the digestive system and include esophageal, liver, gastric, colorectal, and pancreatic cancers. Aerobic glycolysis is a widespread phenomenon among gastrointestinal tumor cells, which poses a major hazard to human health and life. Increasing evidence suggests that aerobic glycolysis can induce and promote the development of gastrointestinal tumors by rapidly providing large amounts of energy and altering the tumor microenvironment. Among them, glucose transporter proteins and key enzymes involved in glycolysis are expressed at higher levels during aerobic glycolysis, and the corresponding signaling pathways and transcription regulatory factors are activated, playing an important role in the occurrence and development of tumors. Additionally, evidence has indicated that aerobic glycolysis plays an essential role in inhibiting the development of immune cells, modifying the population of immune cells present in the surrounding tumor, and promoting the polarization of immune cells. Moreover, drugs and compounds that target essential enzymes and transcription factors associated with glycolysis are known to exhibit anticancer properties.

## 1. Background

Gastrointestinal tumors have a high incidence in the general population and are frequently identified in the later stages of development, with unfavorable prognoses and increased mortality rates. Aerobic glycolysis is a prevalent biological and chemical process in gastrointestinal tumors. Even in the presence of sufficient oxygen, tumor cells prefer to use glycolysis as their primary energy source, a phenomenon known as the Warburg effect 1. Professor Warburg first identified aerobic glycolysis approximately 100 years ago as a marker of hepatocellular carcinoma in rats 1. An increased intake of glucose is a hallmark of aerobic glycolysis, resulting in increased production of intermediates and lactic acid, which provides more adenosine triphosphate (ATP) to tumor cells 2. The accumulation of lactic acid and intermediate products provides a conducive microenvironment for the growth and spread of tumors 3. Glycolysis-related enzymes play an important role in tumor development. Owing to the disparity in glucose absorption rates between malignant and healthy cells, 18F-fluorodeoxyglucose positron emission tomography (^18^F-FDG PET) can be used to diagnose cancer and monitor its progress 4. Glycolysis also promotes tumor invasion, metastasis, angiogenesis, drug resistance, and immune evasion 5. Therefore, aerobic glycolysis plays a crucial role in tumor formation and progression. A comprehensive understanding of the function of aerobic glycolysis in the context of cancer will contribute to future research on targeted therapies. In this article, we discuss the contribution of aerobic glycolysis to the development and progression of gastrointestinal tumors and the underlying mechanisms, including current targeted therapies for aerobic glycolysis.

## 2. Changes in glucose metabolism in gastrointestinal tumors

Aerobic glycolysis is widely considered a key process in tumor growth, mainly because it can rapidly provide energy for tumor cells to grow and proliferate 6 and alter metabolism in the tumor microenvironment 7 thus facilitating suitable conditions for tumor recurrence, metastasis 8, and immune tolerance 9. The multiple biological functions of aerobic glycolysis and its products are gradually being discovered.

### 2.1 Role of glucose transporters in gastrointestinal tumors

Aerobic glycolysis in tumors is an energy-intensive process that requires large amounts of glucose for growth, proliferation, and metastasis. Thus, the rate of glucose uptake via glucose transporters is enhanced to support accelerated metabolism in malignant cells.

Glucose transporter proteins (GLUTs) mediate the initial and most significant step in glucose metabolism, namely the transport of glucose across the plasma membrane in mammalian cells 10. The glucose transporter family comprises 14 proteins that can be divided into three classes based on sequence similarity: class I (GLUTs 1-4, 14), class II (GLUTs 5, 7, 9, and 11), and class III (GLUTs 6, 8, 10, 12, and 13/HMIT). The augmented glucose transport observed in cancerous cells is often linked to the upregulated and dysregulated expression of glucose transporters, with the overexpression of GLUT1 and/or GLUT3 being a typical feature 11. Hypoxic tumor cells upregulate the expression of hypoxia-inducible factor (HIF-1), which plays a pivotal role in mediating the transcriptional regulation of glycolytic genes that carry hypoxia response elements in their promoters, including the glucose transporter proteins GLUT1 and GLUT3 12. The glucose transporter protein GLUT1 is considered the founding member of the GLUT family and enables glucose transport across the hydrophobic cell membrane without requiring energy, thereby reducing the concentration gradient 13. HIF-1 stimulates the expression of GLUT1, which is strongly affinitive to glucose and can transport galactose, mannose, glucosamine, and dehydroascorbic acid (DHA) to promote tumor cell proliferation 14. Moreover, GLUT1 expression is directly associated with tumorigenesis and metastasis 15. Similar to GLUT1, GLUT3 has a strong ability to bind and transport glucose molecules. In addition to glucose, GLUT3 is capable of transporting other molecules such as galactose, mannose, maltose, xylose, and DHA. Tumors with a high glucose demand may utilize GLUT3 as an effective means to enhance glucose uptake into their cells owing to its strong affinity for glucose, ultimately promoting tumor cell proliferation 16. This overexpression has been repeatedly reported in almost all cancer types, suggesting that glucose transporters contribute significantly to cancer cell proliferation and growth. Moreover, the p38-mitogen-activated protein kinase (MAPK) signaling pathway stimulates the use of glucose as an energy source in gastric cancer cells by facilitating the transport of glucose into cells via an increase in the expression of the GLUT4 transporter 17. In colon cancer cells, activation of AKT1 and AKT3 results in the abnormal suppression of miR-125b-5p, which subsequently leads to the upregulation of GLUT5 expression, causing metastasis and drug resistance in colon cancer cells 18. In pancreatic cancer, increased GLUT1 expression is significantly correlated with poorer prognosis, larger tumor size, and lymph node metastasis 19.

### 2.2 Glycolysis produces ATP for tumor cells

**Figure 1:**
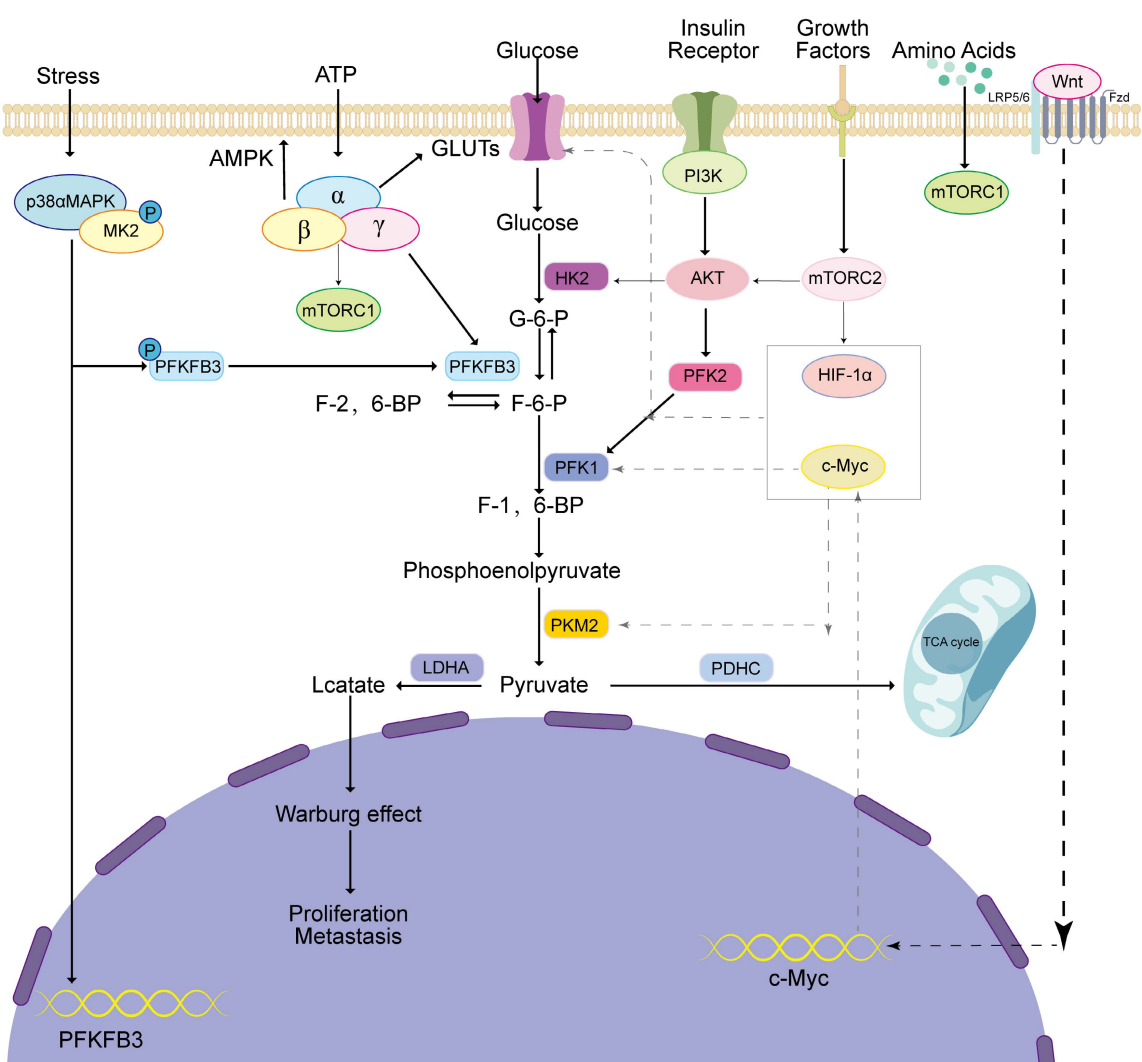
Metabolism of glucose in tumor cells involves several steps. Firstly, glucose is taken into the cells by GLUT and then transformed by key glycolytic enzymes such as HK2, PFKFB3, PFK1, and PKM2. This transformation generates pyruvate, which is eventually converted into lactate by LDHA. This process of lactate production promotes the Warburg effect, which ultimately aids tumor progression. Simultaneously, if pyruvate is prevented from entering the mitochondria and participating in the TCA cycle, it can cause mitochondrial dysfunction and further promote tumor development.

Glucose is metabolized by tumor cells in two ways. First, when oxygen is sufficient, glucose generates pyruvate in the cytoplasm, which enters the mitochondria for oxidative phosphorylation, where it is finally metabolized into carbon dioxide and water, releasing a large amount of energy 20. Second, the conversion of pyruvate to lactate is facilitated by the enzyme lactate dehydrogenase in the cytoplasm under hypoxic conditions 21 (Figure 1).

Glycolysis generates a significant quantity of lactic acid that builds up in the tumor microenvironment to form an acidic environment and reduce the pH of the microenvironment 22. The resulting acidic microenvironment promotes tumor growth and metastasis 23, angiogenesis 24, and immunosuppression 25. In particular, lactic acid-induced GPR81 activation promotes tumor growth 26. Lactic acid promotes tumor metastasis and invasion by remodeling the extracellular matrix, increasing cell activity, and enhancing epithelial-mesenchymal transition (EMT) 27. Lactic acid promotes angiogenesis by stimulating macrophages to secrete vascular endothelial growth factors (VEGF), enhancing endothelial cell migration and vascular morphogenesis, and recruiting circulating vascular precursor cells 28. Lactic acid can upregulate inhibitory molecules, produce immunosuppressive cytokines, and downregulate costimulatory molecules to induce immune tolerance in tumors 29.

The swift multiplication and dissemination of cancerous cells throughout the body require an abundant and rapid supply of ATP, which is generated by aerobic glycolysis in tumor cells 30. The regulation of aerobic glycolysis is influenced by three critical enzymes: hexokinase (HK), phosphofructokinase (PFK), and pyruvate kinase (PK), and the equilibrium enzyme fructose-bisphosphatase 31. The rate of tumor growth and proliferation, along with prognosis, is influenced by changes in the function or expression of these enzymes or isozymes.

#### 2.2.1 Hexokinase

HK is the initial bottleneck enzyme in the glycolytic pathway, irreversibly catalyzing the conversion of glucose into glucose-6-phosphate, and exists in the human body mainly as four isozymes: HK1, HK2, HK3, and HK4. There is significant upregulation of HK2 expression in many malignancies, and its high expression is associated with cell proliferation, invasion, metastasis, recurrence, and poor prognosis 32. Multiple studies indicate that elevated HK2 expression is associated with resistance to radiotherapy 33.

HK domain-containing 1 (HKDC1), which is significantly elevated in hepatocellular carcinoma, stimulates the multiplication and migration of malignant liver cells by upregulating the Wnt/β-catenin pathway 34. In gastric cancer, mesenchymal stem cells exhibit a G6PD-NF-KB-HGF signaling pathway that promotes growth and spread 35. HK2 is also involved in apoptosis and autophagy. For example, mitochondrial membrane stability is promoted by the interaction between HK2 and voltage-dependent anion channels located in the mitochondria, which prevents the binding of pro-apoptotic factors, thereby inhibiting apoptosis 36. HK2 can stimulate autophagy under glucose-depleted conditions and glycolytic activity even when adequate glucose is present; thus, HK2 can switch between autophagy and glycolysis depending on the amount of glucose 37.

#### 2.2.2 Phosphofructokinase

PFK1 plays a pivotal role as the second rate-controlling enzyme in the glycolytic pathway, irreversibly facilitating the transformation of fructose-6-phosphate to fructose-1,6-bisphosphate by utilizing ATP as a co-substrate 38. The human body contains three different isoforms of PFK1, PFKM in the muscles, PFKL in the liver, and PFKP in the blood, in different proportions in different tissues. PFK1 is inhibited by the negative feedback activity of its downstream products, which regulate the pace of the glycolytic pathway through their controlling actions, including the production of phosphoenolpyruvate, lactate, citrate, and ATP 39. Notably, 6-Phosphofructo-2-Kinase/Fructose-2,6-Bisphosphatase 3 (PFKFB3) catalyzes the conversion of fructose-6-phosphate (F-6-P) to fructose-2,6-bisphosphate (F-2,6-BP), the strongest metabotropic stimulator of PFK1 with the highest potency 40 which significantly increases the activity of PFK1 and enhances glycolysis. PFK2 and PFKFB can control the levels of fructose-2,6-bisphosphate through their regulatory functions 41, which is important for regulating glycolysis, cell proliferation, and metastasis.

PFK is widely expressed in gastrointestinal tumors. Overexpression of PFKFB3 causes epithelial-mesenchymal transition (EMT) by increasing the expression of Snail and Twist, which enhances the migration and invasion abilities of gastric cancer cells 42. Human colorectal adenoma and adenocarcinoma tissues exhibit significant PFKFB3 expression, and its overexpression has been linked to lymph node metastasis, intravascular cancer thrombosis, and TNM staging in patients with sporadic colorectal cancer 43. The expression of PFKFB3 and PFKFB4 is detectable in pancreatic cancer cells, which display a significant hypoxia-induced response mediated by HIF-1 44.

#### 2.2.3 Pyruvate kinase

PK regulates the last step of glycolysis by irreversibly converting phosphoenolpyruvate into pyruvate, thereby controlling the rate at which glucose is metabolized to produce energy. PK consists of four isomers: hepatic PKL, blood PKR, muscle PKM1, and PKM2. PKM2 is significantly more highly expressed in malignant tumors 45 and facilitates the transformation of phosphoenolpyruvate into pyruvate, generating ATP, thus increasing the rate of glycolysis 46. PKM2 enters the nucleus and promotes the conversion of DNA into RNA molecules for specific genes, leading to positive feedback regulation of glycolysis 47. PKM2 enhances tumor cell growth and hinders autophagy via the JAK/STAT3 pathway in hepatocellular carcinoma 48. In addition, PKM2 facilitates the formation of new blood vessels in tumors by modulating HIF-1, ultimately leading to the activation of NF-κB 49. Cross-talk between the mechanistic target of rapamycin (mTOR)/PKM2 and STAT3/c-myc signaling pathways modulates the acid-base balance of the microenvironment and the metabolic state in gastric cancer 50.

### 2.3 Aerobic glycolysis-related key signaling pathways

**Figure 2:**
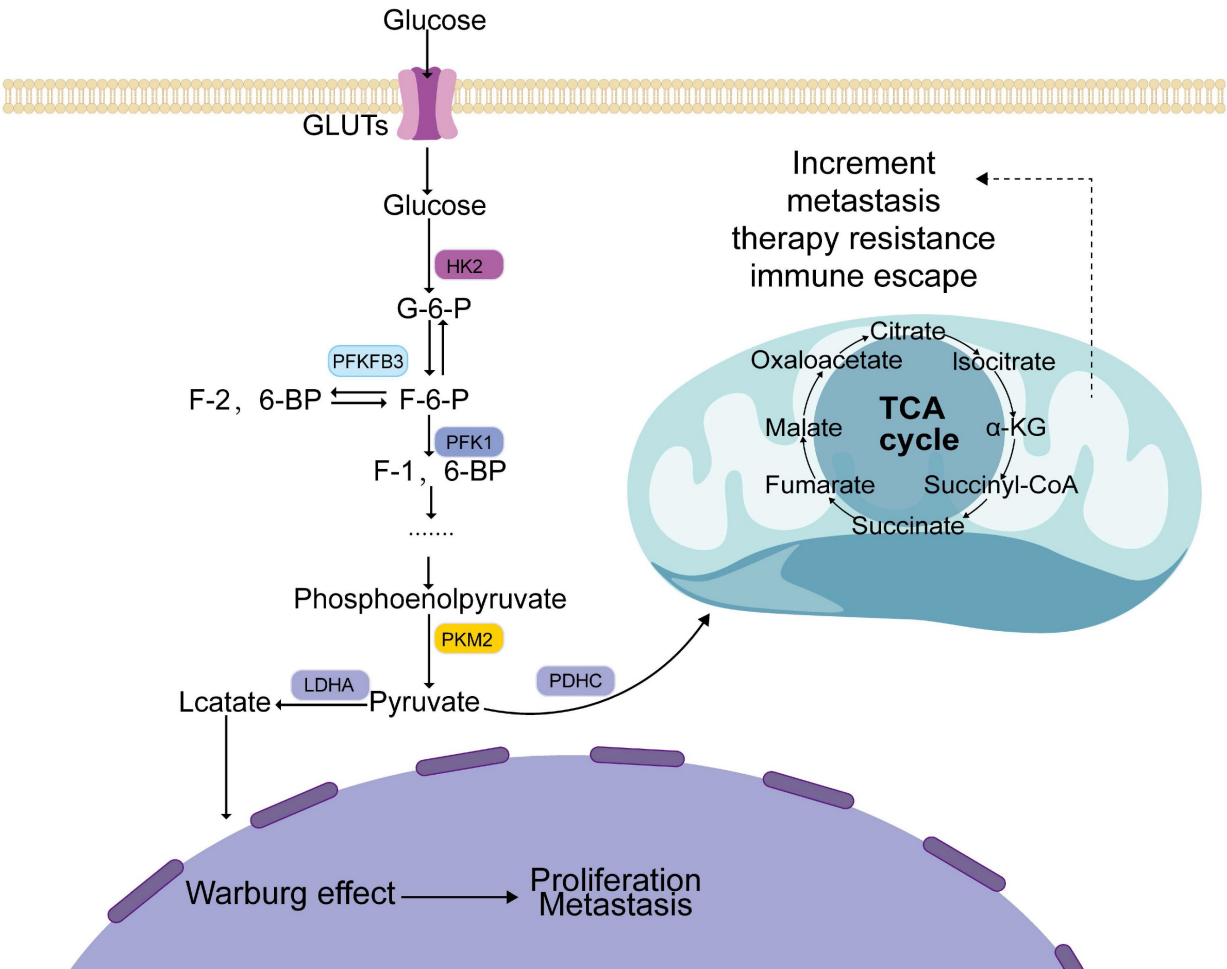
Regulation of the main pathways of aerobic glycolysis. MAPK, AMPK, PI3K, mTORC, and Wnt pathways regulate aerobic glycolysis and promote tumor occurrence and development through the expression of glucose transporters, glycolytic key enzymes, and transcription factors.

Several signaling pathways contribute significantly to the development and progression of tumors 51, metastatic recurrence 52, development of drug resistance 53, and EMT 54. The AMP-activated protein kinase (AMPK) 55, phosphatidylinositol 3-kinase/protein kinase B (PI3K/AKT) 56, mTOR 57, MAPK 58, and Wnt 59 signaling pathways can affect aerobic glycolysis in tumor cells (Figure 2).

#### 2.3.1 AMPK pathway

AMPK, a protein kinase that phosphorylates serine/threonine residues, shows a high degree of evolutionary conservation and acts as a sensor to maintain energy homeostasis 60. AMPK is composed of three subunits, with a catalytic α subunit and a regulatory β/γ subunit forming a heterotrimeric complex. AMPK can directly sense the AMP:ATP ratio and is rapidly activated when ATP levels are reduced, as in the case of glucose starvation 61, metabolic inhibition 62, or muscle contraction 63. Recent studies have demonstrated that AMPK mediates the suppression of tumor cell growth 64.

AMPK directly inhibits mTORC1 activity by phosphorylating TSC2 (tuberous sclerosis complex 2) 65 and RAPTOR (a subunit of mTORC1) 66. mTORC1 is a critical regulator of cell growth and protein synthesis, and its overactivation is closely linked to the development of various cancers. By inhibiting mTORC1, AMPK reduces tumor cell proliferation and survival. AMPK activates multiple tumor suppressor proteins, such as p53, p27, and pRb, which play key roles in inducing cell cycle arrest 67. In addition to regulating the cell cycle, AMPK also activates ULK1 (Unc-51-like autophagy activating kinase 1), a critical regulator of autophagy 68. Moreover, under conditions of glucose deficiency, AMPK collaborates with the Hippo tumor suppressor pathway to phosphorylate and inactivate YAP (Yes-associated protein) 69. Moreover, since abnormal activation of the hedgehog pathway is implicated in the development of many cancers, AMPK targeting of GLI1 reduces hedgehog signaling, further contributing to the suppression of tumor cell proliferation 70.

Furthermore, AMPK hinders the tumorigenesis of esophageal cancer via an AMPK/FOXO3a/BIM-dependent mechanism 71. In hepatocellular carcinoma, AMPK promotes the degradation of HIF-1α via the autophagy-lysosome pathway involving AMPK/ULK1 72.

#### 2.3.2 PI3K/AKT pathway

The PI3K/AKT pathway induces glucose metabolism in tumors. Phosphorylation of intracellular inositol lipids by PI3Ks enables them to function as lipid kinases that regulate signaling and intracellular vesicle transport. PI3Ks can be categorized into three distinct groups based on their substrate specificity and structural characteristics. The main function of Class I PI3Ks is to generate 3-phosphatidylinositol lipids, which act as signaling molecules to directly activate downstream signal transduction pathways, whereas Class II PI3Ks govern a range of cellular processes, including proliferation, migration, primary cilia function, glucose consumption, survival, and angiogenesis. Class III PI3Ks are crucial for autophagy, endosomal transport, and phagocytosis. The enzyme AKT is a serine/threonine kinase that exists in three forms, termed isoforms, namely AKT1, AKT2, and AKT3. It is a crucial downstream effector of the PI3K signaling pathway and is involved in various essential cellular processes such as cancer cell viability, cell cycle initiation, and glucose consumption 73.

AKT is directly activated by PI3K 74. The phosphorylation of AKT downstream of PI3K activates PFK2, which stimulates the synthesis of fructose-2,6-bisphosphate, the primary agonist of PFK1, and enhances glycolysis 75. The PI3K/AKT signaling pathway promotes an increase in GLUT1 expression 76, and the PI3K/AKT pathway can facilitate the transport of this molecule from the cytoplasm to the plasma membrane 77. Activation of the insulin receptor substrate-2 stimulates the PI3K/AKT pathway and insulin-sensitive GLUT4 in the plasma membrane to promote glucose uptake, thus transactivating insulin action 78. Therefore, the activation of the PI3K/AKT signaling pathway can increase the levels of specific enzymes and transporters that play a role in glycolysis.

In liver cancer, the upregulation of microRNA-17-5p can decrease PTEN gene expression 79, which is a significant inhibitor of the PI3K/AKT pathway 80, thereby activating the PI3K/AKT signaling pathway and promoting glycolysis. In colon cancer, the PI3K/AKT pathway activates mTOR and regulates the expression of downstream targets 4EBP1 and p70S6K, thus promoting the expression of genes that facilitate the cell cycle 81. During angiogenesis in colon cancer, the activation of the PI3K/AKT pathway can be triggered by various signals 82, including vascular endothelial growth factor (VEGF), and oversees critical phases by modifying certain downstream proteins through the addition of phosphate groups to promote angiogenesis 83. In gastric cancer, PDZK1 deficiency leads to the activation of the PI3K/AKT signaling pathway, which is linked to unfavorable patient outcomes 84. In addition, activation of the PI3K/AKT pathway prompts gastric cancer cells to acquire traits resembling those of stem cells 85. Therefore, targeting the PI3K/AKT signaling pathway has gained considerable interest as a potential therapeutic strategy.

#### 2.3.3 mTOR pathway

mTOR plays a crucial role in helping cells progress through different stages of their life cycle, divide to form new cells, regulate cell survival and metabolism, and control the cytoskeleton in response to amino acids, stress, oxidative stress, energy demands, and growth factors. The mTOR kinase family is composed of three main parts: mTOR1, mTOR2, and mTOR3, of which both mTOR1 and mTOR2 have been linked to cancer 86.

mTORC1 is a group of proteins that work together, including Raptor, PRAS40, mLST8 (or GβL), and Deptor, all of which help regulate the activity of mTOR 87. By activating mTORC1, cells can shift their glucose metabolism from the ordinary oxidative phosphorylation pathway to glycolysis, thus facilitating cellular proliferation and growth, while enhancing the levels of HIF1α expression 88. mTORC1 also induces the pentose phosphate pathway, as well as the biosynthesis of sterols and lipids, and regulates the expression of G6PD 89. In addition, mTORC2 plays a role in reorganizing the actin cytoskeleton and promoting cell migration; its activity is not affected by rapamycin 90, and its role in protein synthesis, protein maturation, autophagy, and metabolic regulation has been noted 90.

In hepatocellular carcinoma, mTORC1 downregulates the expression of NEAT1/2 and inhibits NEAT1/2-mediated biogenesis of paranuclear spots, thereby promoting mRNA splicing and the expression of critical glycolytic enzymes. Moreover, mTORC1 signaling is involved in aberrant metabolism, hepatocarcinogenesis, and the response to mTORC1-targeted therapy in hepatocellular carcinoma 91. Colorectal cancer often involves dysregulation of the mTOR pathway, leading to activation of the AKT/mTOR axis and increased expression of c-Myc and HIF-1α; the former promotes glycolysis, and the latter accelerates the metabolic rate in colon cancer cells 92. In addition, mTOR can increase glutamine flux, and thus glutaminase activity, by upregulating c-myc 93. Through this pathway, oncogenic AKT and mTOR trigger protein synthesis to stimulate the expansion and reproduction of colorectal cancer cells while inhibiting the apoptosis of cancer cells. In esophageal cancer, abnormal activation of the mTOR pathway upregulates the production of HIF-1α, accelerates cellular metabolism, and increases the expression of PKM2, which induces aerobic glycolysis 94. Therefore, the mTOR pathway is essential for aerobic glycolysis in gastrointestinal tumors.

#### 2.3.4 MAPK pathway

The MAPK pathway is among the earliest identified intracellular signaling pathways and is involved in the evolution of multiple physiological processes. The MAPK pathway is involved in the cellular metabolic shifts during tumorigenesis. The MAPK family includes ERK, JNK, and p38-MAPK, which regulate the transcription of immediate early genes (IEGs) in response to cellular stress 95. When MAPK is activated, the phosphorylated p38α-MAPK forms a complex with MAPK-MK2 to promote transcription, protein synthesis, cellular receptor expression, and cytoskeletal changes, thereby altering cell survival and apoptosis 96. A recent study has indicated that PFKFB3 is an important target of MAPK-MK2; therefore, the MAPK pathway may affect glycolysis 97.

p38, PFKFB3 phosphorylates MK2 at Thr334, leading to transcription and direct activation of PFKFB3. MK2 increases PFKFB3 transcription by promoting the phosphorylation of Ser103 in SRF, which activates SRE in the promoter region of PFKFB3. In addition, phosphorylated MK2 promotes direct phosphorylation of PFKFB3 at Ser461, leading to increased PFKFB3 function and formation of fructose-2,6-bisphosphate, which triggers PFK1 metabotropic activation, thus enhancing the rate of glycolysis 97.

In hepatocellular carcinoma, the extracellular matrix regulates YAP by affecting the MAPK signaling pathway, which contributes to the reprogramming of cancer metabolism 98, including glycolysis, and increased expression of YAP strengthens aerobic glycolysis and the migration of tumor cells 99. In addition, by modulating the MAPK/ERK pathway, the hypoxia-induced lncRNA NPSR1-AS1 promotes both proliferation and glycolysis in hepatocellular carcinoma cells 58.

#### 2.3.5 Wnt pathway

The Wnt signaling pathway is a biological mechanism that has remained unchanged throughout the course of evolution and plays a key role in embryonic development, stem cell maintenance, and wound healing. The Wnt signaling pathway is divided into a β-linked protein-dependent pathway (typical pathway) and a non-β-linked protein-dependent pathway (atypical pathway). Even though the two pathways have similarities, the non-classical pathway has not been extensively studied, and research has mostly focused on the classical pathway, which has β-linked protein as the main effector. Wnt proteins control a variety of cellular functions and activities, including cell multiplication, differentiation, movement, and the generation of new stem cells. The development of several types of cancer is linked to dysregulation of the Wnt signaling pathway. Studies have demonstrated various functional effects of oncogenic Wnt signaling, including increased proliferation, induction of EMT, promotion of angiogenesis and migration, and enhanced cell survival.

Wnt/β-catenin signaling raises glucose uptake and inhibits mitochondrial oxidative phosphorylation 100. Wnt upregulates the expression of pyruvate carboxylase, an enzyme that converts pyruvate to oxaloacetate, to promote cancer cell proliferation 100. Wnt5B, a ligand of Wnt, regulates *c-Myc*, which negatively affects mitochondrial function 101, and positively correlated with MCL1, a mitochondrial regulator 102. c-Myc plays a crucial role in regulating cancer cell metabolism, particularly in aerobic glycolysis, and also acts as a transcription factor that mediates the Wnt/β-catenin pathway's control over this process 103.

In gastric cancer, the tumor cells exhibit elevated levels of LRP5 expression, and high expression of LRP5 increases the energy supply to tumor cells by triggering the typical Wnt/β-catenin signaling pathway and increasing aerobic glycolysis by upregulating it 104. In hepatocellular carcinoma, autophagy promotes glucose uptake and lactate production by upregulating the expression of monocarboxylate transporter 1 (MCT1) and activating Wnt/β-catenin signaling 59.

### 2.4 Transcriptional regulation of glucose metabolism

Transcription factors are essential signal transduction components that are involved in cellular gene expression. They recognize specific DNA sequences and bind to specific response elements located in genomic regions responsible for promoting or enhancing gene expression. Transcription factors are considered the end effectors of cellular signaling pathways. Notably, transcription factors are essential for modulating the Warburg effect, and several examples of transcription factors that regulate glycolysis are listed in Table 1.

#### 2.4.1 HIF-1

Activation of HIF-1 increases with tumor growth 105. GLUTs 106 and other glycolytic enzymes 107 are upregulated by HIF-1 either directly or indirectly, thereby promoting tumor cell proliferation. Other stimuli, such as insulin, insulin-like growth factor 1, epidermal growth factor, and angiotensin II, have also been shown to increase HIF-1 levels in cells 108.

The initial stage of glucose metabolism in mammalian cells is plasma membrane glucose transport, which is controlled by GLUT and serves as a major rate-limiting factor 109. A typical feature of malignant cells is an increase in glucose transport caused by dysregulated expression of glucose transporters. Overexpression of GLUT1 and/or GLUT3 is common in these types of cells 109. In hypoxic tumor cells, HIF-1 expression is increased, which mediates the transcriptional regulation of genes related to glycolysis that have hypoxia response elements in their promoters, such as GLUT1 and GLUT3 110.

#### 2.4.2 c-Myc

c-Myc is a helix-loop leucine zipper transcription factor that binds the chaperone protein Max dimer to specific DNA sequences, thereby activating genes in trans. It regulates various cellular functions, including cell growth, differentiation, apoptosis, protein synthesis, cell adhesion, and energy metabolism.

c-Myc directly regulates key enzymes and GLUTs involved in glycolysis, most notably GLUT1, HK2, PFKM, and enolase 1 (ENO1) 111. Therefore, gene expression can be promoted by c-Myc, which increases glucose transport, the catabolism of monosaccharides, and pyruvate, and their conversion to lactate. Under normoxic conditions, c-Myc promotes glucose oxidation and lactate production. However, under hypoxic conditions, c-Myc synergistically induces pyruvate dehydrogenase kinase 1 (PDK1) with HIF-1, thereby inhibiting mitochondrial respiration and facilitating the transformation of glucose into lactate 112.

In pancreatic cancer, activation of c-Myc-lactate dehydrogenase A (LDHA) stimulates glucose utilization, lactate production, proliferation, migration, and invasion of pancreatic cancer cells 113. In colorectal cancer, increased expression of LDHA, PKM2, and GLUT1, associated with glycolysis, was observed in c-Myc-overexpressing HCT116 cells, and the ECAR assay showed that the interference of far upstream element-binding protein 1 was reversed by c-Myc overexpression, resulting in the inhibition of glycolysis 114. The expression of c-Myc is remarkably high in gastric cancer, and its binding to the promoter of PDK1 regulates the expression of PDK1, which inhibits PDH activity by phosphorylating PDH, thereby reducing pyruvate conversion into acetyl coenzyme A in the tricarboxylic acid (TCA) cycle. This reduces mitochondrial oxidative phosphorylation, thus promoting pyruvate conversion into lactate, promoting aerobic glycolysis in gastric cancer cells, and reducing the pH of the tumor microenvironment via aerobic glycolysis by LDHA 115.

#### 2.4.3 p53

p53 acts as a tumor suppressor and regulates key cellular processes such as proliferation, invasion, metastasis, apoptosis, stemness, metabolic reprogramming, cell cycle arrest, and DNA repair. Glucose metabolism in cancer cells is impacted by the activation of p53, which hinders the growth of a highly aggressive tumor phenotype 116.

p53 is involved in several glycolytic processes; it can inhibit glucose transport by directly suppressing the transcription of GLUT1 and GLUT4 117 and can also repress NF-κB, leading to a decrease in GLUT3 expression 118. In addition, the direct induction of ras-related glycolysis inhibitors and calcium channel regulators (RRAD) can hinder the translocation of GLUT1 to the plasma membrane by inhibiting glucose transport 119. p53 inhibits glycolysis by indirectly repressing GLUT4 expression, and indirectly inhibits glucose uptake by repressing the insulin receptor promoter (INSR) 120 and downregulating insulin receptor expression. Furthermore, p53 upregulates parkin, which ubiquitinates PKM2 and decreases glycolytic rates 121. It also inhibits MCT1 by repressing lactate transport, which leads to lactate accumulation and reduced glycolysis in cancer cells 122. In addition, p53 downregulates pyruvate dehydrogenase kinase 2 and upregulates parkin, resulting in increased PDH activity 123. p53 induces the production of TIGAR, which dephosphorylates F-2,6-P2 to F6P 124. F-2,6-P2 is a strong transactivator of PFK1. Therefore, TIGAR inhibits glycolysis. P53 also affects glycolysis by downregulating HK2 at the transcriptional level and participating in the degradation of phosphoglycerate during metastasis 125. Taken together, these findings show that the activation of p53 can aid in reversing the Warburg effect 126.

In colon cancer cells, IC261 reduced the expression of p53 and TIGAR, upregulated GLUT1 mRNA expression, and promoted aerobic glycolysis 127. In pancreatic cancer cells, missense mutations in p53 prevent the nuclear translocation of the glycolytic enzyme 3-phosphoglyceraldehyde dehydrogenase (GAPDH) and stabilize its cytoplasmic localization, thus encouraging glycolysis in cancerous cells and obstructing apoptosis mediated by nuclear GAPDH 128. In hepatocellular carcinoma, TIGAR and SCO2 transcription is controlled by TCF19 and p53, which enhance energy production and increase resistance to stress by improving mitochondrial function 129.

## 3. Glycolysis mediates cancer immunity

**Figure 3:**
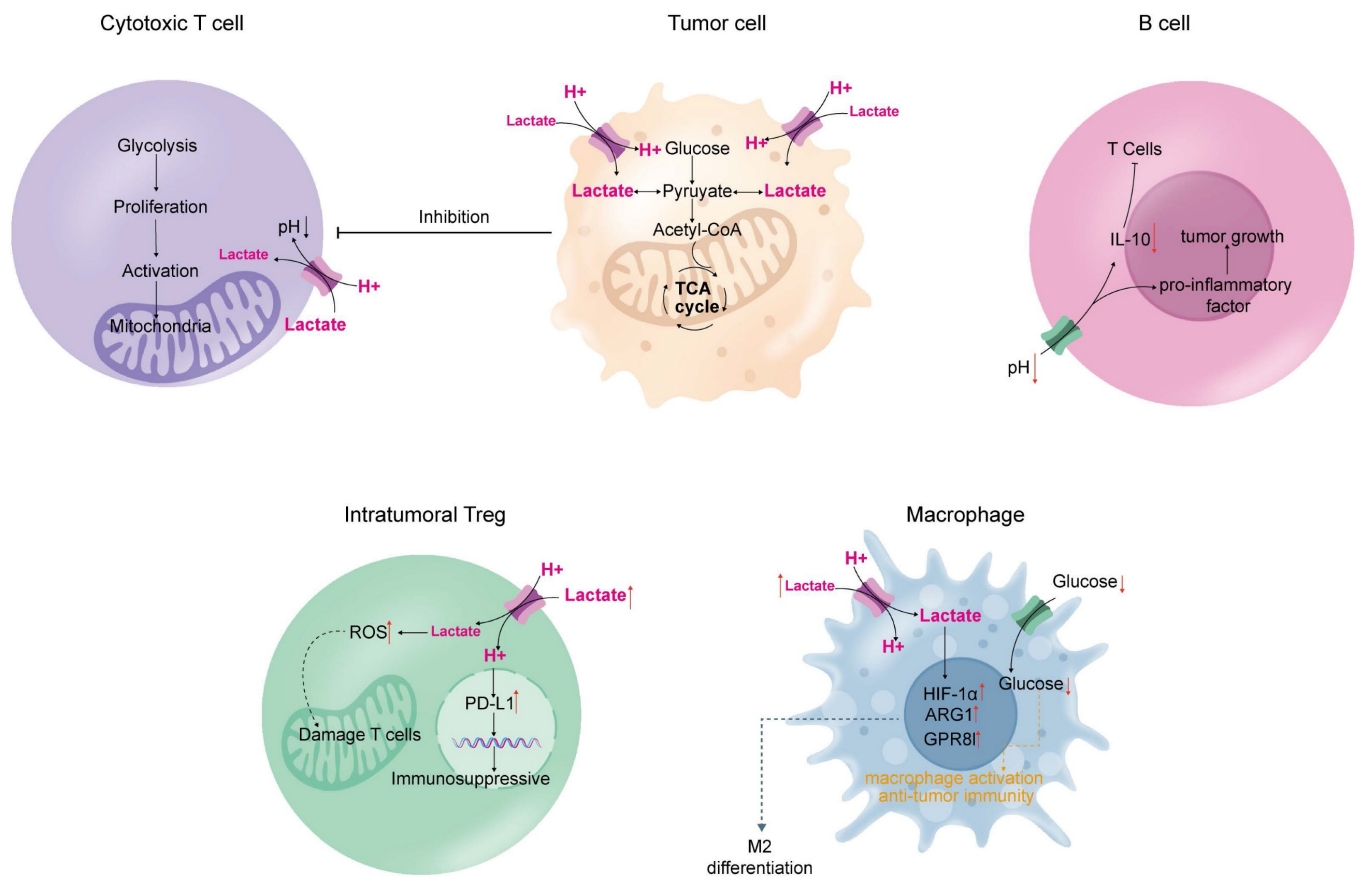
Impact of the tumor microenvironment on immune cells. Tumor cells produce a large amount of lactate through aerobic glycolysis, which is released into the microenvironment and affects the lactate concentration gradient inside immune cells. This inhibits the normal immune functions of cytotoxic T cells, macrophages, B cells, and intratumoral T cells, leading to immune deficiency in tumors, ultimately resulting in tumor progression.

Aerobic glycolysis in tumors leads to metabolic alterations that can impair immune function 130. This involves three main mechanisms: (1) tumor cells compete for nutrients with growing and activated immune cells during proliferation; (2) byproducts of aerobic glycolysis in tumor cells inhibit the function of immune cells and modulate immune performance; and (3) genes and signaling pathways that regulate glycolysis produce factors and metabolites associated with immune suppression (Figure 3).

### 3.1 T-cell

Large amounts of lactate from aerobic glycolysis are released into the tumor microenvironment, creating a concentration gradient that prevents T cells within the microenvironment from releasing the lactate produced during their activation and proliferation 131. High lactate levels in T cells inhibit their growth and activation 132.

Acidity, hypoxia, and lack of nutrients are distinguishing features of the tumor metabolic environment, which hinder T cells from exerting effective anti-tumor immunity and developing long-term immune memory 130. The build-up of lactic acid in the synovial fluid of patients with rheumatoid arthritis has been shown to hinder T-cell mobility 133, and this phenomenon can be observed in tumors. In addition, T cell adaptation to the tumor microenvironment may lead to mitochondrial deficiency and dysfunction, directly resulting in T cell immune dysfunction 134.

### 3.2 Regulatory T cells (Treg)

The hypoxic and acidic tumor microenvironment not only reduces anti-tumor immunity by blunting effector T-cell responses but also drives immune escape by encouraging the enlistment and function of pro-tumor immune cells that suppress the immune system (e.g., Tregs) 135. Involved in maintaining immune balance, shielding against autoimmune conditions, and preventing excessive inflammation, Tregs are a distinct type of CD4+ T cells 136. However, increased Treg ratios have been identified in the tumor microenvironment across various tumor types and are associated with a poor prognosis (pancreatic ductal adenocarcinoma 137, non-small cell lung cancer 138, ovarian cancer 139 and glioblastoma 140).

Basal levels of reactive oxygen species (ROS) are significantly higher in Tregs than in other T-cell subsets 141, and increased production of ROS by Tregs may be harmful to effector T-cells 142. In addition, lactate uptake by Tregs upregulates the expression of programmed death ligand 1 (PD-L1), which largely influences Treg-mediated immunosuppression and the efficacy of PD-L1 blockade therapy 143.

### 3.3 Macrophages

Macrophages are classified as M1-like inflammatory and M2-like regulatory types 144. M1 macrophages eliminate cancer cells, whereas M2 macrophages promote the growth of cancer cells 145. As macrophages grow and mature, their metabolism shifts to glycolysis and lactate production. Interestingly, high extracellular lactate levels polarize macrophages into immunosuppressive M2-like macrophages 146. Moreover, lactate can drive M2 polarization by mediating the expression of HIF1-α, thus causing an impact on the development of macrophages that results in the M2-like characteristic 147. The uptake of lactate by macrophages induces arginase 1 (ARG1), which converts M1 macrophages into M2 macrophages 148. ARG1 hinders the initiation and reproduction of T cells 149. Lactate can also drive signals from the GPR81 receptor on macrophages, promoting the production of immunosuppressive agents with M2-like characteristics 150.

### 3.4 B-cell

B cells are the second type of adaptive immune cells found in the tumor microenvironment 151. In an acidic microenvironment due to the presence of a tumor, B cells exhibit tumor growth-promoting properties 152. B cells produce or induce other cells to produce interleukin-10 to suppress T cells 153, 154. PD-L1^+^ B cells act on natural immune cells to promote immune tolerance 155. In addition, B cells directly drive cancer cell growth by producing proinflammatory cytokines 156.

## 4. Aerobic glycolysis as a therapeutic target in the treatment of gastrointestinal tumors

**Figure 4:**
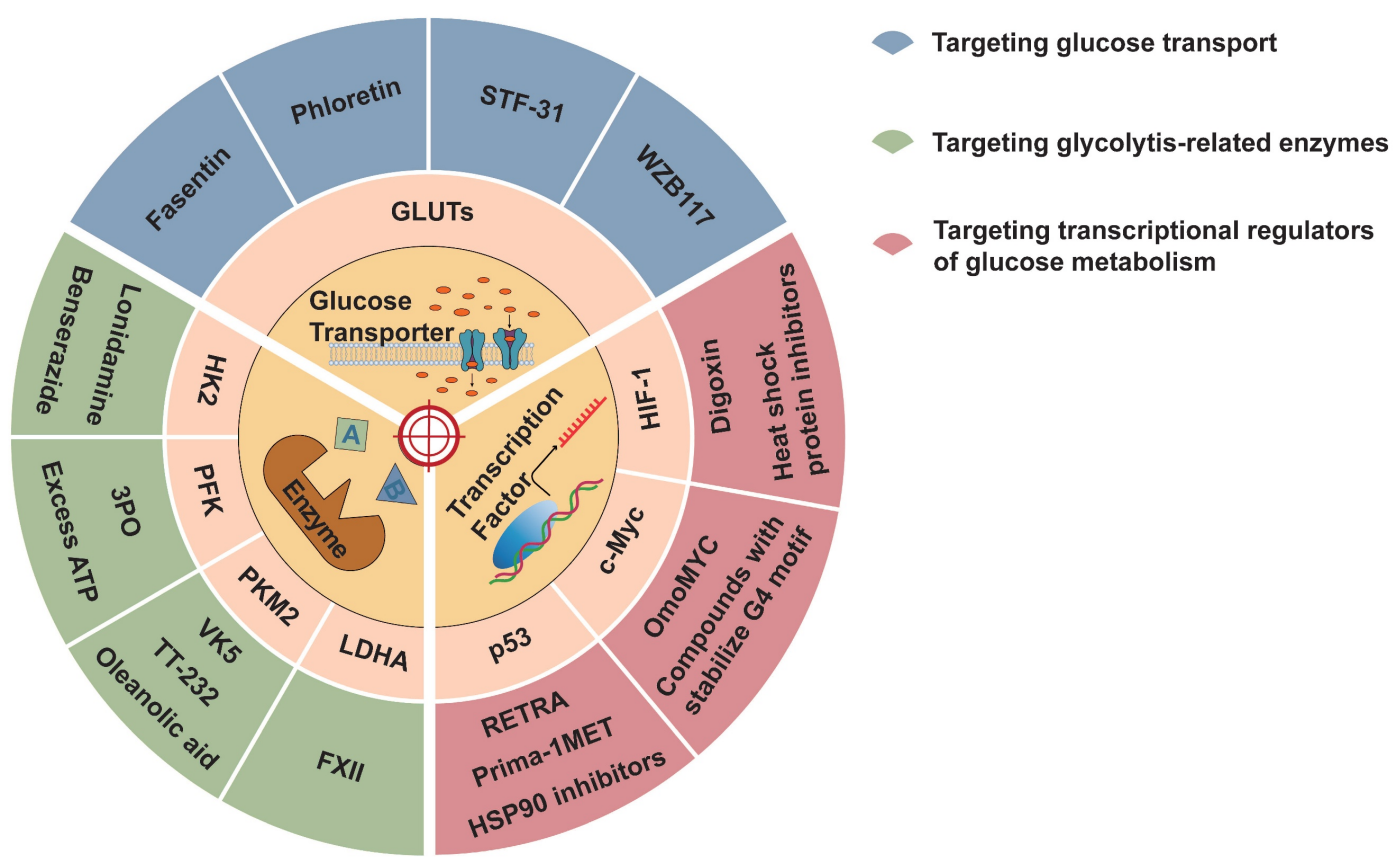
Inhibitors of aerobic glycolysis. Aerobic glycolysis inhibitors can be divided into three categories: those targeting the glucose transporter, glycolysis key enzymes, and transcription factors.

Aerobic glycolysis can promote tumor development, drug resistance, and suppression of antitumor immunity. Therefore, it is possible to interfere with different mechanisms of aerobic glycolysis to inhibit tumor occurrence and development. Several drugs and inhibitors targeting aerobic glycolysis are described below (Figure 4 and Table 2).

### 4.1 Targeting glucose transport

The transport of glucose into tumor cells via glucose transporters is the initial stage of glucose metabolism. Targeted glucose transporter therapy inhibits tumor occurrence and development. Drugs that target GLUT1 include fasentin, phloretin, STF-31, and WZB117. Fasentin can inhibit glucose transport and reduce resistance to caspase activation, thereby improving drug efficacy against tumors 157. Phloretin inhibits the expression of GLUT1 and GLUT2 and activates p53 to suppress tumors by inhibiting the growth of colon cancer cells 158. STF-31 selectively inhibits GLUT1, which hinders tumor growth and reduces tumor size with few side effects 159. Similarly, WZB117 is a highly effective GLUT1 inhibitor that can irreversibly hinder the transport of glucose by GLUT1, thus inhibiting tumors from performing aerobic glycolysis and reducing the ATP concentration in cells, leading to cessation of the cell cycle 160. Research findings indicate that targeting glycolysis can impede the proliferation of cancerous cells both in vivo and in vitro, and act synergistically with cisplatin and paclitaxel 161. In addition, GLUT3 inhibition effectively inhibits EMT in colon cancer cells 162.

### 4.2 Targeting glycolytic-related enzymes

In most cases, glycolysis-related enzymes are overexpressed in tumors. Targeting these enzymes can effectively inhibit the Warburg effect, thereby controlling tumor growth and metastasis. Therapeutic modalities that target HK2 include HK2 inhibitors, such as Lonidamine, 3-Bromopyruvate, and benserazide, which have the potential to restrict the development, multiplication, and programmed cell death of cancerous cells 163.

Excess ATP and 3-(3-Pyridinyl)-1-(4-pyridinyl)-2-propen-1-one(3PO) 164 can inhibit the activity of PFK, thus hindering tumor growth. In addition, TT-232, VK3, VK5, and Compound 3 inhibit tumor cell glycolysis by inhibiting PKM2 165. Oleanolic acid can convert PKM2 into PKM1 to inhibit the function of PKM2 166. FXII, which inhibits LDHA, depletes intracellular ATP, leading to inhibition of tumor growth and cell death 167.

### 4.3 Targeting transcriptional regulators of glucose metabolism

Digoxin can inhibit HIF-1 expression as well as tumor growth and proliferation, and heat shock protein inhibitors can degrade HIF-1 168. OmoMYC can form homologous dimers that bind to DNA and inhibit c-Myc-max from binding to DNA and c-Myc expression 169. Additionally, inhibitors of BET proteins and compounds that stabilize G4 motifs in the Myc promoter can reduce Myc expression and achieve tumor suppression 170. Prima-1MET restores wild-type p53 function and inhibits tumor growth 171. Studies have shown that HSP90 inhibitors 172 and HDAC inhibitors 173 can induce degradation of mutant p53 to achieve therapeutic effects in tumors. RETRA disrupts the p53-p73 complex and inhibits tumor growth via p53 mutations through a pathway that relies on p73 for its operation 174. The inhibition of kinases involved in the G2/M checkpoint, such as PKC, can inhibit the survival pathway of p53-mutated cells and lead to the death of p53-mutated cells 175.

## 5. Conclusions and perspectives

A distinguishing feature of cancer is aerobic glycolysis, commonly referred to as the Warburg effect. Aerobic glycolysis is a common mechanism that supports the proliferation and metastasis of GI tumors. Glucose transporters (GLUT1, GLUT3, etc.), key enzymes (HK, PFK, PK), signaling pathways (PI3K/AKT, mTOR, AMPK, MAPK, and Wnt), and some transcription factors (HIF-1, c-Myc, and p53) are involved in the aerobic glycolysis of gastrointestinal tumors. The byproducts of aerobic glycolysis, including lactic acid, alter the tumor microenvironment, which affects the normal function of immune cells in the tumor microenvironment, leading to weakened antitumor immunity and increased drug resistance. Therefore, aerobic glycolysis is a potential therapeutic target.

This paper reviews the roles of key enzymes in glycolysis, the regulation of related signaling pathways and transcription factors, and the alterations and roles of immune cells within the tumor milieu. These factors are key to the occurrence of metabolic rearrangement, apoptosis, the cell cycle, and drug resistance, thus largely affecting the growth, invasion, and metastasis of gastrointestinal tumors. Thus, aerobic glycolysis is a potential therapeutic target for the treatment of gastrointestinal tumors.

## Figures and Tables

**Table 1:** Transcription factors HIF-1, c-Myc, and p53 regulate the role of glycolytic enzymes in cancer

Transcription factors	Expression in cancer	Target	Effect	References
HIF-1	Positive	GLUT1 GLUT3	Up-regulate Up-regulate	10,12,109 10,12,109
c-Myc	Positive	GLUTs HK2 PFKM ENO1 PDK1 PKM2	Up-regulate Up-regulate Up-regulate Up-regulate Up-regulate Up-regulate	111,114 111 111 111 112 114
p53	Negative	GLUT1 GLUT3 GLUT4 PKM2 MCT1 TIGAR	Down-regulate Down-regulate Down-regulate Down-regulate Down-regulate Down-regulate	117,119,127 118 117,120 121 122 124,126,127,129

**Table 2:** Glycolytic inhibitors that modulate glycolytic metabolism

Target	Inhibitors	Mechanisms of action	References
GLUTs	Fasentin Phloretin STF-31 WZB117	Inhibits glucose transport and reduces drug resistance Inhibits the expression of GLUTs and activates p53 Inhibits GLUT1 Inhibits GLUT1 and cell cycle; inhibits GLUT3 and EMT	157 158 159 160
HK2	Lonidamine 3-Bromopyruvate Benserazide	Inhibits HK2 Inhibits HK2 Inhibits HK2	163 163 163
PFK	Excess ATP 3PO	Inhibits the activity of PFK Inhibits the activity of PFK	164 164
PKM2	TT-232 VK3 VK5 Compound 3 Oleanolic acid	Inhibits PKM2 Inhibits PKM2 Inhibits PKM2 Inhibits PKM2 Inhibits PKM2; converts PKM2 into PKM1	165 165 165 165 166
LDHA	FXII	Inhibits LDHA; depletes intracellular ATP	167
HIF-1	Digoxin Heat shock protein inhibitors	Inhibits HIF-1 expression Degrades HIF-1	168 168
C-Myc	OmoMYC BET protein inhibitor Compounds with stabilize G4 motif	Inhibits the binding of Myc-max to DNA and myc expression Reduce myc expression Reduce myc expression	169 170 170
p53	Prima-1MET HSP90 inhibitors HDAC inhibitors RETRA PKC	Restores wild-type p53 function Induce degradation of mutant p53 Induce degradation of mutant p53 Disrupts the p53-p73 complex Inhibit the survival pathway of p53-mutated cells	171 172 173 174 175

## Abbreviations

AMPK: AMP-activated protein kinase
ATP: Adenosine triphosphate
^18^F-FDG PET: 18F-fluorodeoxyglucose positron emission tomography
GLUT: Glucose transporter
HIF-1: Hypoxia-inducible factor
DHA: Dehydroascorbic acid
EMT: Epithelial-mesenchymal transition
HK: Hexokinase
MAPK: Mitogen-activated protein kinase
VEGF: Vascular endothelial growth factor
HKDC1: HK domain-containing 1
mTOR: Mechanistic target of rapamycin
PFKFB3: 6-Phosphofructo-2-Kinase/Fructose-2:6-Biphosphatase
3 PFK: Phosphofructokinase
PK: Pyruvate kinase
Treg: Regulatory T cells
PRAS40: Proline rich AKT substrate 40 kDa
MCT1: Monocarboxylate transporter 1
ENO1: Enolase 1
PDK1: Pyruvate dehydrogenase kinase 1
INSR: Insulin receptor promoter
ROS: Reactive oxygen species
TNF-α: Tumor necrosis factor-α
